# Supplementary material for: Mitochondrial ATP synthase as a direct molecular target of chromium(III) to ameliorate hyperglycaemia stress
Source: Nat Commun. 2023 Mar 28;14:1738. doi: 10.1038/s41467-023-37351-w (PMC10050403; doi:10.1038/s41467-023-37351-w)
Supplement: Supplementary file 1 — Supplementary Information [file 41467_2023_37351_MOESM1_ESM.pdf]

SUPPLEMENTARY INFORMATION

Mitochondrial ATP synthase as a direct molecular target of chromium(III) to ameliorate hyperglycaemia stress

Haibo Wang,[1]† Ligang Hu,[1,2]† Hongyan Li,[1]† Yau-Tsz Lai,[1] Xueying Wei,[1] Xiaohan Xu,[1] Zhenkun Cao,[1] Huiming Cao,[3] Qianya Wan,[4] Yuen-Yan Chang,[1] Aimin Xu,[5] Qunfang Zhou,[2] Guibin Jiang,[2] Ming-Liang He,[4] and Hongzhe Sun[1*]

[1]Department of Chemistry, State Key Laboratory of Synthetic Chemistry, CAS-HKU Joint Laboratory of Metallomics on Health and Environment, The University of Hong Kong, Hong Kong S.A.R., P. R. China.

[2]State Key Laboratory of Environmental Chemistry and Ecotoxicology, Research Center for Eco-Environmental Sciences, Chinese Academy of Sciences, Beijing, P.R. China.

[3]Institute of Environment and Health, Jianghan University, Wuhan 430056, P.R. China.

[4]Department of Biomedical Science, City University of Hong Kong, Hong Kong, P.R. China.

[5]Department of Pharmacology and Pharmacy, and State Key Laboratory of Pharmaceutical Biotechnology, The University of Hong Kong, 21 Sassoon Road, Hong Kong, P.R. China.

†These authors contributed equally to this work.

*Correspondence and request materials should be addressed to H.S. (E-mail: hsun@hku.hk).

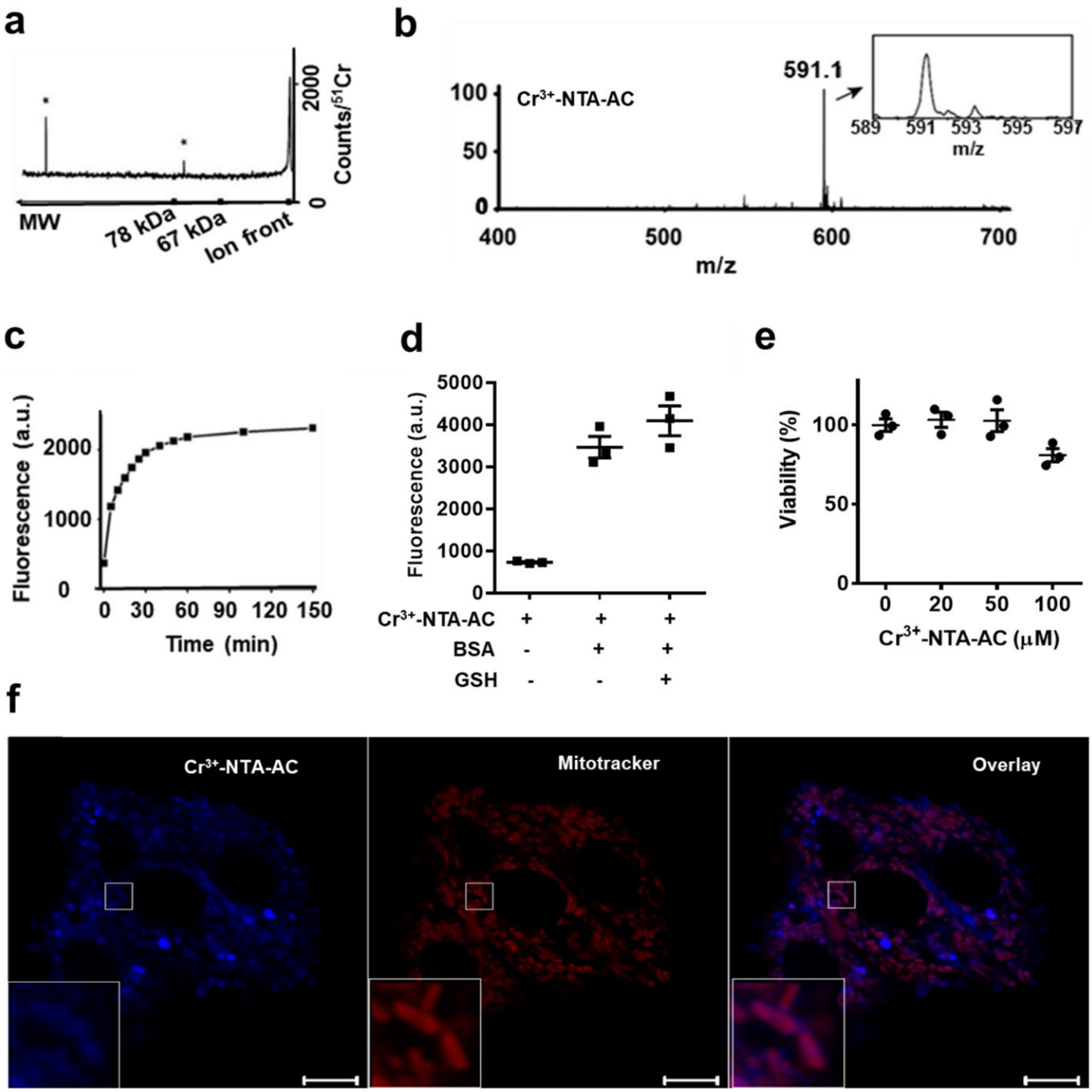

Supplementary Fig. 1 | Visualizing the distribution of Cr^{3+} via fluorescent labeling. (**a**) Representative profile of Cr^{3+}-binding proteins in the lysate of Cr^{3+} ($CrCl_3$) treated HepG2 measured by ICP-MS coupled with column-type gel electrophoresis under native condition (n = 5). The asterisks are ghost peaks resulted from fluctuation of the instrument, which often occurs as noises during the ICP-MS analysis. (**b**) MS confirms the formation of Cr^{3+}-NTA-AC. The peak at 591.1 corresponding to $[M-2H-K]^-$ (calcd. m/z 590.3). The Cr^{3+}-NTA-AC was synthesized by reacting equimolar amounts of fluorescent ligand with Cr^{3+} (as $CrCl_3$) in buffered aqueous solution. (**c**) Time-dependent fluorescence response of Cr^{3+}-NTA-AC (5 μM) upon incubation with bovine serum albumin (BSA, 50 μM). A.u. denotes arbitrary unit. The fluorescence increased rapidly to ~ 4 times in 30 min and reached a plateau in 1 hr with ~ 5-fold enhancement, demonstrating that Cr^{3+}-NTA-AC can rapidly label the Cr^{3+} associated proteins. (**d**) Fluorescence "turn-on" of Cr^{3+}-NTA-AC (1 μM) was initiated by *ca.* 5 folds upon binding to bovine serum albumin (BSA) (1 μM). n = 3; mean ± SEM. The fluorescence response would not be deviated under physiological level of GSH (5.6 mM). BSA was selected to validate the feasibility of Cr^{3+}-NTA-AC to label proteins. (**e**) Cytotoxicity test on Cr^{3+}-NTA-AC. Negligible cytotoxicity of Cr^{3+}-NTA-AC at concentration up to 50 μM was noted. n = 3; mean ± SEM. (**f**) Representative confocal microscopic imaging of live HepG2 cells labeled with 50 μM Cr^{3+}-NTA-AC and MitoTracker® red (n = 5) under normal glucose (5.6 mM) condition. Co-localization of the fluorescence of Cr^{3+}-NTA-AC (blue) and MitoTracker® red was observed (pink), indicating that Cr^{3+}-associated proteins are localized in mitochondria. Scale bars: 10 μm. Source data are provided as a Source Data file.

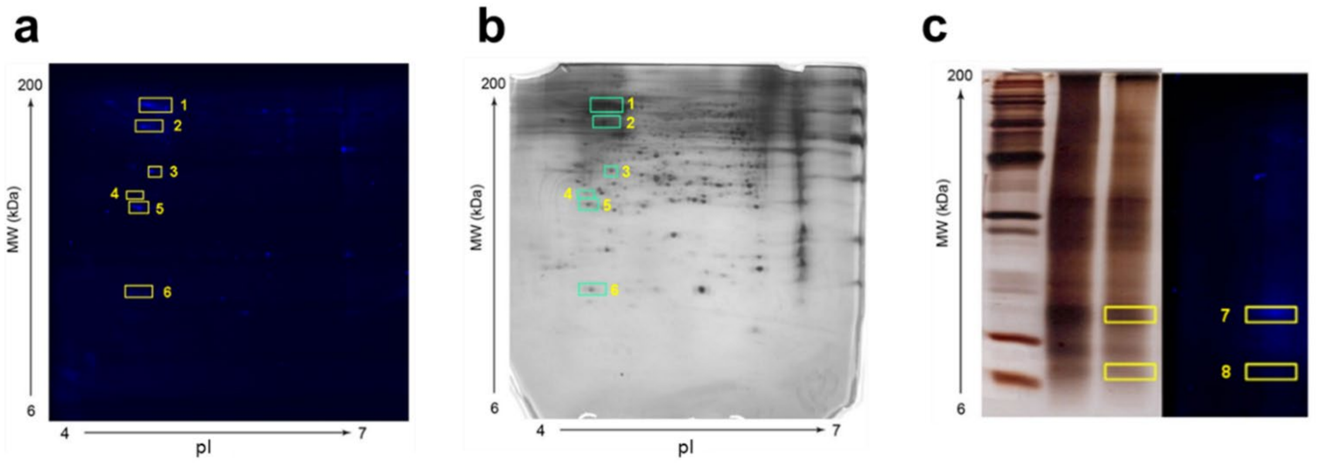

Supplementary Fig. 2 | Separation and identification of Cr^{3+}-associated proteins by gel electrophoresis. (**a**, **b**) Two-dimensional electrophoresis (2-DE) of soluble protein fraction of HepG2 cells treated with 50 μM Cr^{3+}-NTA-AC. Fluorescent (a) and silver stained (b) images of 2-DE. (**c**) SDS-PAGE gel separation of insoluble fraction of HepG2 cells. The lit-up protein spots and bands are highlighted in boxes. The spots were excised and subjected to protein identification by MALDI-TOF-MS. In both 1D SDS-PAGE and 2-DE study, the fluorescent images were overlapped with the silver-stained images to confirm the position of the lit-up spots and then further excising for protein identification. Fluorescent spots that could not overlap well with a protein spot by silver-staining were not submitted for protein identification. Given the high sensitivity of fluorescence labeling, it could be possible that the quantity of protein identified was not sufficient to give positive signals by silver staining, or capable of submitting for MS identification after excising from the gel. One representative result of three independent experiments is shown (**a**, **b**, **c**). Source data are presented in the figure.

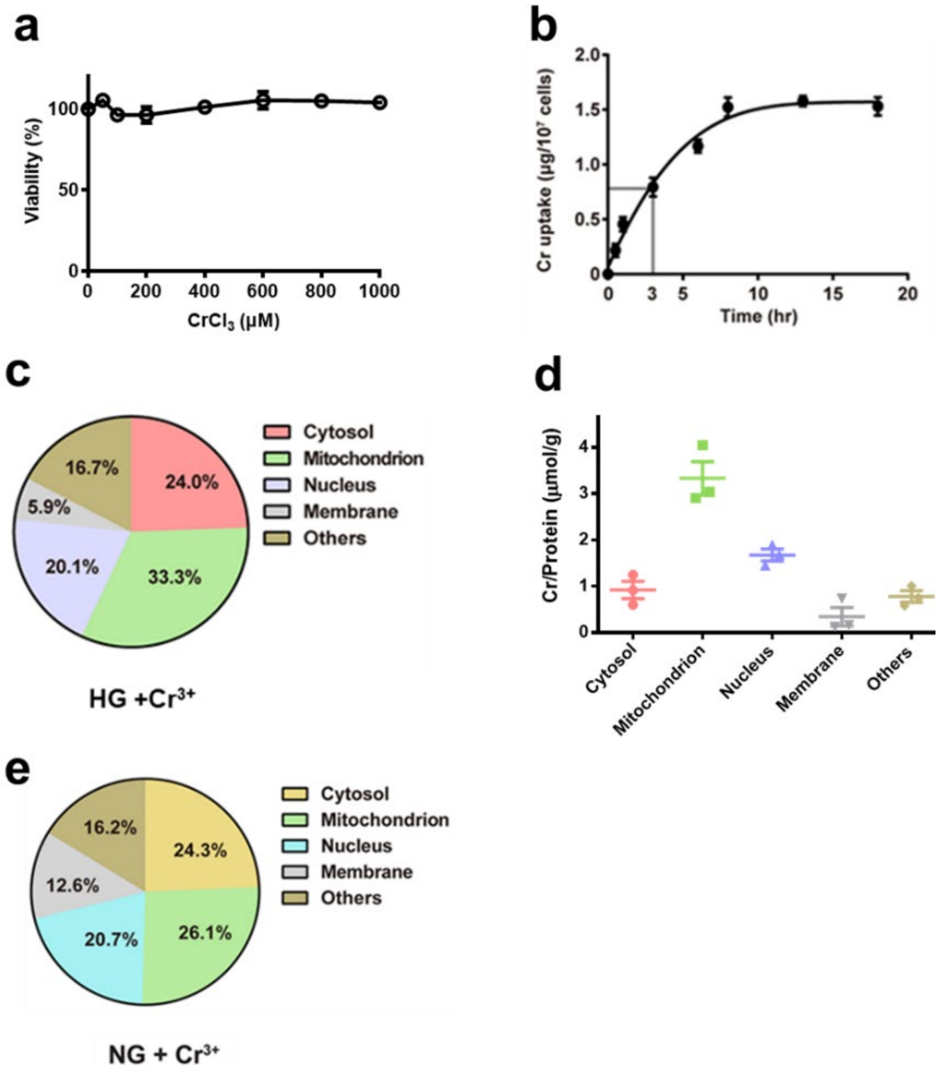

Supplementary Fig. 3 | Chromium(III) cellular uptake and distribution. (**a**) Cytotoxicity test of $CrCl_3$. n = 3; mean ± SEM. Negligible cytotoxicity of $CrCl_3$ at concentration up to 1000 μM was noted. (**b**) Time-dependent uptake of Cr^{3+} in HepG2 cells after treatment with 100 μM $CrCl_3$. n = 3; mean ± SEM. Cr^{3+} distribution in different components of HepG2 cells treated with 100 μM $CrCl_3$ for 8 hrs under hyperglycaemia (**c**) and normal glucose condition (**e**). (**d**) Cr^{3+} to protein ratio in different cellular components of HepG2 cells. n = 3; mean ± SEM. Source data are provided as a Source Data file.

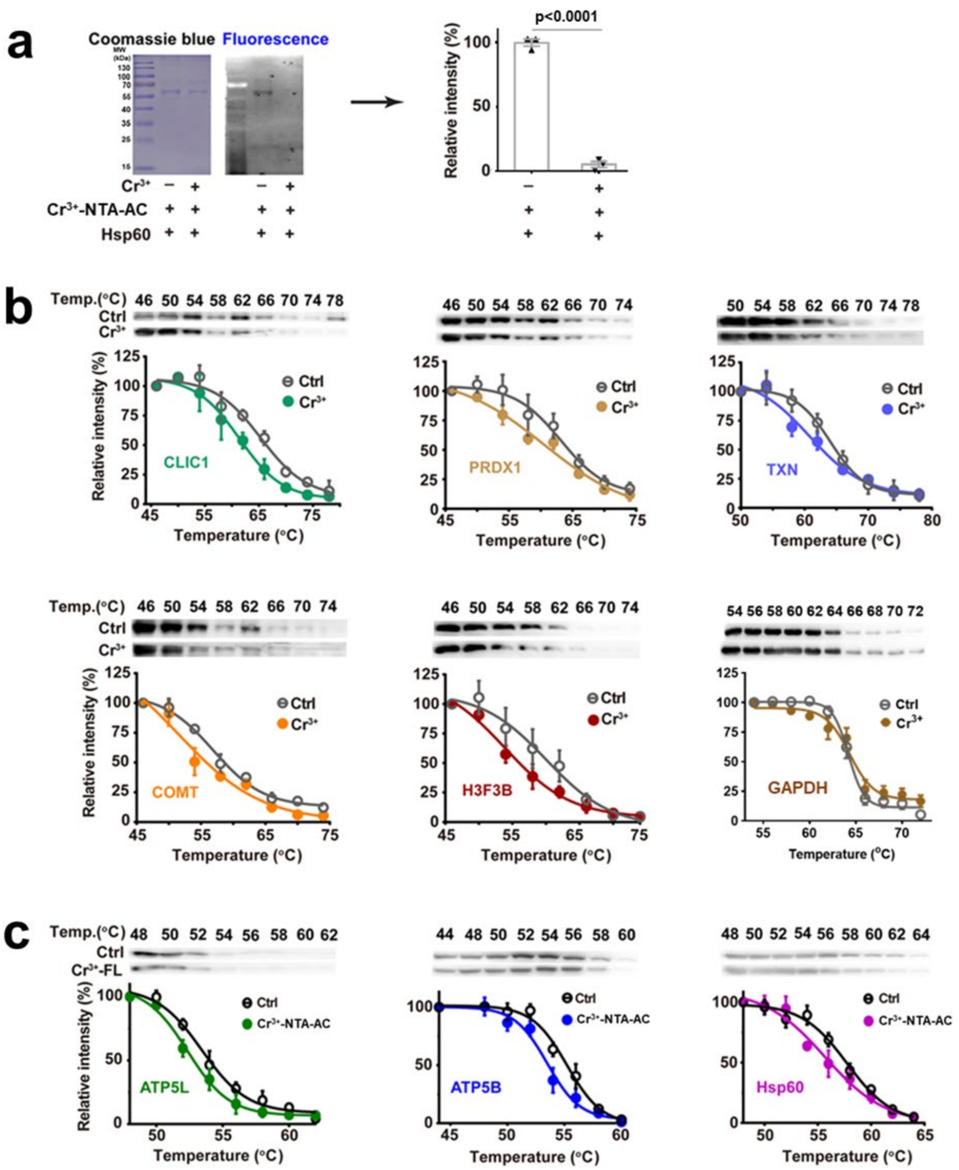

Supplementary Fig. 4 | Validation of binding of $CrCl_3$ or Cr^{3+}-NTA-AC to the identified proteins. (**a**) SDS-PAGE of Hsp60 with Coomassie Blue staining and fluorescence image. n = 3; mean ± SEM. Two-sided Student's *t* test. (**b**) Cellular thermal-shift assays (CETSA) demonstrated that Cr^{3+} binds to CLIC1, PRDX1, TXN, COMT, and H3F3A in cellulo but not GAPDH. HepG2 cells were treated with 100 μM Cr^{3+} (as $CrCl_3$) under hyperglycaemia conditions and the thermal changes of the identified Cr^{3+}-binding proteins were monitored. n = 3; mean ± SEM. (**c**) CETSA demonstrated that Cr^{3+}-NTA-AC binds to Hsp60, ATP5B and ATP5L in cellulo. n = 3; mean ± SEM. Similar thermal destabilization of these proteins by Cr^{3+}-NTA-AC in HepG2 cells was observed under identical conditions with three mitochondrial proteins Hsp60, ATP5B and ATP5L as showcases, indicating that Cr^{3+}-NTA-AC resembles $CrCl_3$ for the identification of protein targets. One representative western blot result of three independent experiments is shown (a, b, and c). Source data are provided as a Source Data file.

a

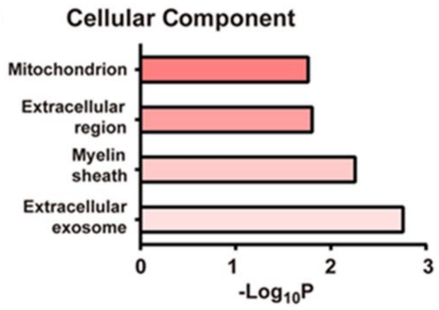

GO ID	GO term	Count	*P*-value	Protein associated
GO.0005739	Mitochondrion	5	0.0173	ATP5B,COMT, Hsp60,PRDX1,TXN
GO.0005576	Extracellular region	7	0.0157	ATP5B,ATP5L,COMT, H3F3A,Hsp60,PRDX1,TXN
GO.0043209	Myelin sheath	3	0.00554	ATP5B,Hsp60,PRDX1
GO.0070062	Extracellular exosome	7	0.00174	ATP5B,ATP5L,COMT, H3F3A,Hsp60,PRDX1,TXN

b

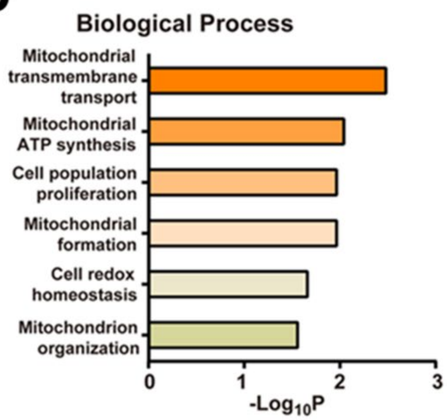

GO ID	GO term	Count	*P*-value	Protein associated
GO:1990542	Mitochondrial transmembrane transport	3	0.0033	ATP5B,ATP5L,Hsp60
GO:0042776	Mitochondrial ATP synthesis	2	0.0091	ATP5B,ATP5L
GO:0008283	Cell population proliferation	4	0.0108	H3F3A,Hsp60,PRDX1,TXN
GO:0042407	Mitochondrial formation	2	0.0108	ATP5B,ATP5L
GO:0045454	Cell redox homeostasis	2	0.0218	PRDX1,TXN
GO:0007005	Mitochondrion organization	3	0.0277	ATP5B,ATP5L,Hsp60

Supplementary Fig. 5 | Bioinformatics analysis. (**a**) Gene ontology (GO) enrichment analysis on the targeted proteins demonstrates that mitochondrion is the most relevant cellular component (GO: 0005739, $p = 1.7 \times 10^{-2}$), with five proteins (PRDX1, Hsp60, ATP5B, TXN, and COMT) located in the mitochondrion. (**b**) The mitochondrial ATP synthesis coupled proton transport (GO: 0042776, $p = 9.1 \times 10^{-3}$) is one of the most related biological processes, with the proteins (ATP5B and ATP5L) directly related to ATP synthase process. Source data are presented in the figure.

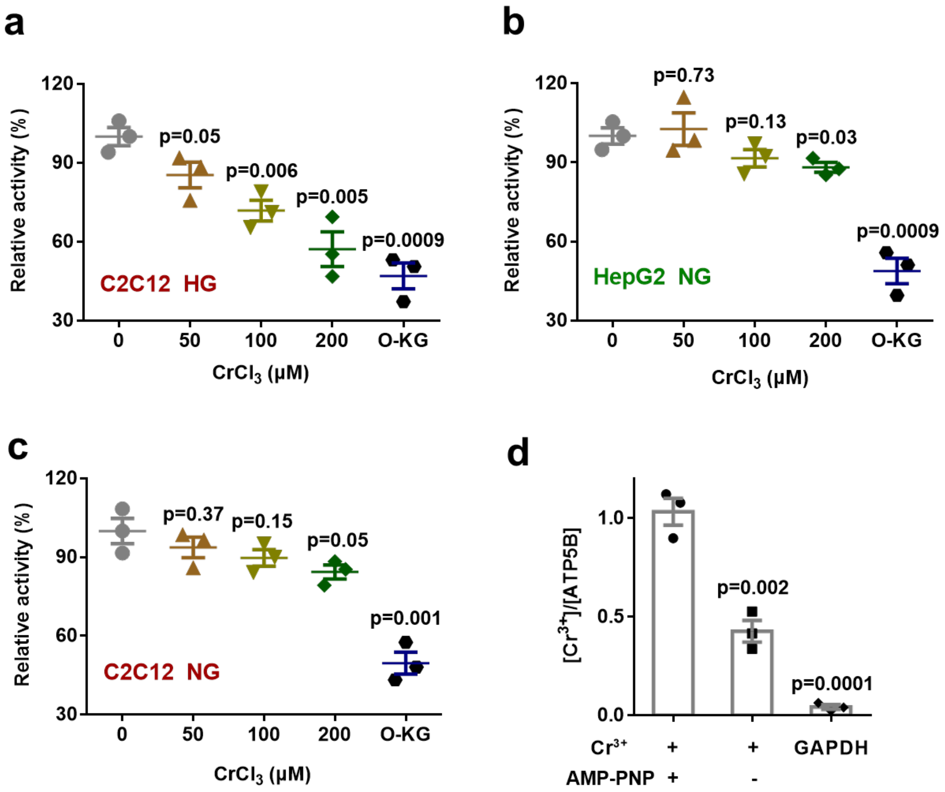

Supplementary Fig. 6 | ATP synthase activity measurement. (**a**) The effect of Cr^{3+} on ATP synthase activity of C2C12 cells under hyperglycemia condition (HG). n = 3; mean ± SEM. Two-sided Student's *t* test. (**b**) The effect of Cr^{3+} on ATP synthase activity of HepG2 cells under normal glucose condition (NG). n = 3; mean ± SEM. Two-sided Student's *t* test. (**c**) The effect of Cr^{3+} on ATP synthase activity of C2C12 cells under normal glucose condition (NG). n = 3; mean ± SEM. Two-sided Student's *t* test. ATP5B inhibitor Octyl-α-ketoglutarate (O-KG) is used as control. (**d**) The effect of nucleotide on Cr^{3+} binding to ATP5B as determined by ICP-MS. n = 3; mean ± SEM. Two-sided Student's *t* test. GAPDH is used as a control. Source data are provided as a Source Data file.

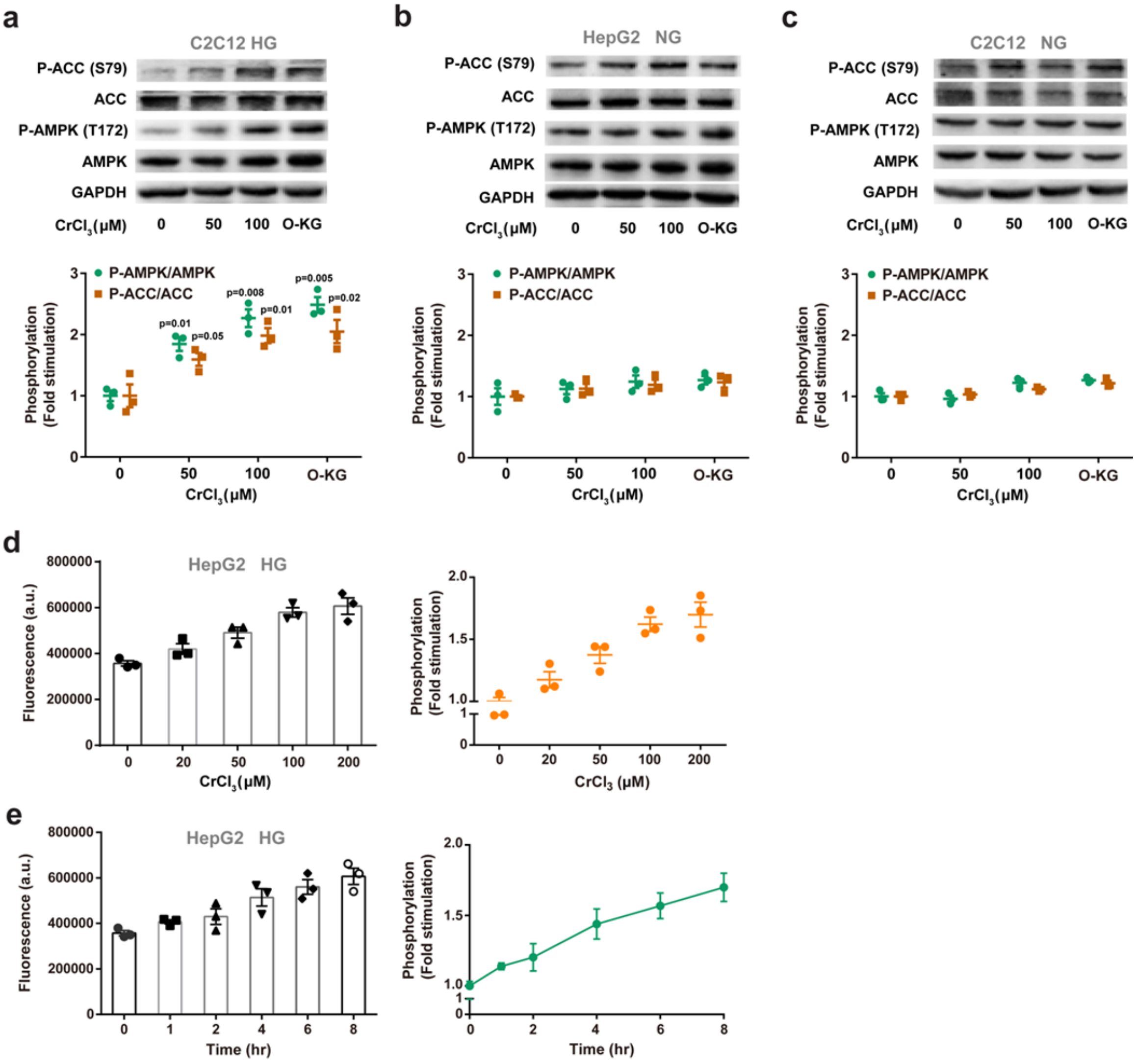

Supplementary Fig. 7 | Cr^{3+} activates AMPK under hyperglycaemia condition. Effect of Cr^{3+} treatment on AMPK and ACC activation under hyperglycemia condition (**a**) C2C12 cells and normal glucose condition (**b**) C2C12 cells (**c**) HepG2 cells. n = 3; mean ± SEM. Two-sided Student's *t* test. (**d**) Dose-dependent activation of Cr^{3+} on AMPK in HepG2 cells under hyperglycemia condition measured by AMPK activity assay kit, in which AMPK activity is represented by the contents of P-AMPK with an antibody-based fluorescence approach. n = 3; mean ± SEM. (**e**) Time-dependent activation of Cr^{3+} on AMPK. n = 3; mean ± SEM. HG, high glucose (40 mM). NG, normal glucose (5.6 mM). One representative western blot result of three independent experiments is shown (**a**, **b**, and **c**). Source data are provided as a Source Data file.

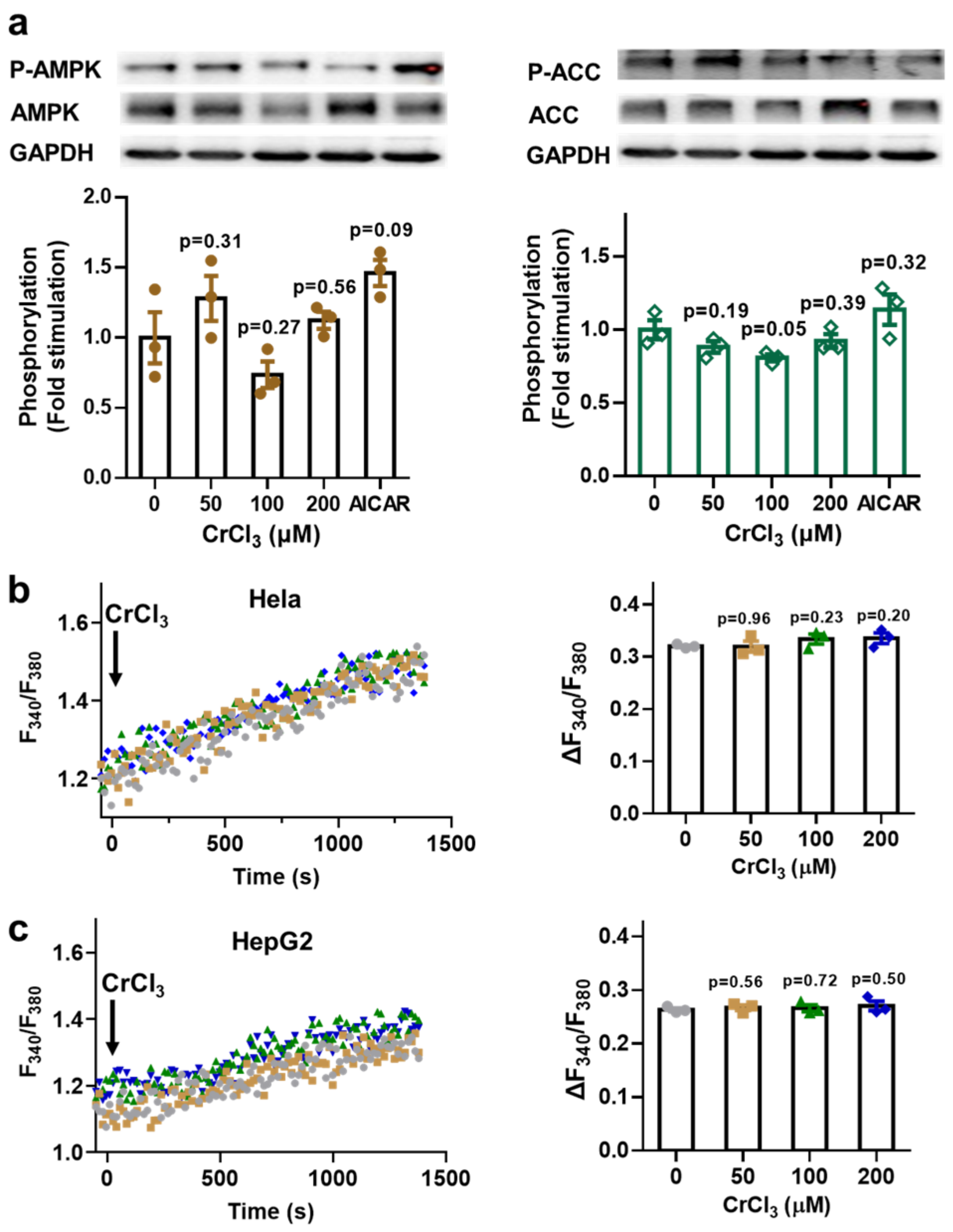

Supplementary Fig. 8 | LKB1 is involved in Cr^{3+}-mediated activation of AMPK. (**a**) Effect of Cr^{3+} treatment on AMPK and ACC activation in Hela cells under hyperglycemia condition. n = 3; mean ± SEM. Two-sided Student's *t* test. Intracellular Ca^{2+} levels in Hela cells (**b**) and HepG2 cells (**c**) after treatment of various concentration of $CrCl_3$. n = 3; mean ± SEM. Two-sided Student's *t* test. One representative western blot result (a) and real time fluorescence curve (b and c) of three independent experiments is shown. Source data are provided as a Source Data file.

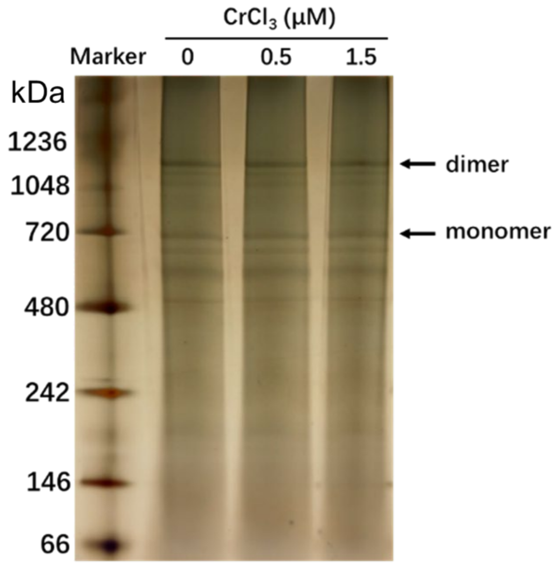

Supplementary Fig. 9 | Effect of Cr^{3+} treatment on ATP synthase oligomerization state. Digitonin protein extracts of mitochondria isolated from Hela cells were separated by 4-16% blue native page (BNP). One representative result of three independent experiments is shown. Source data are presented in the figure.

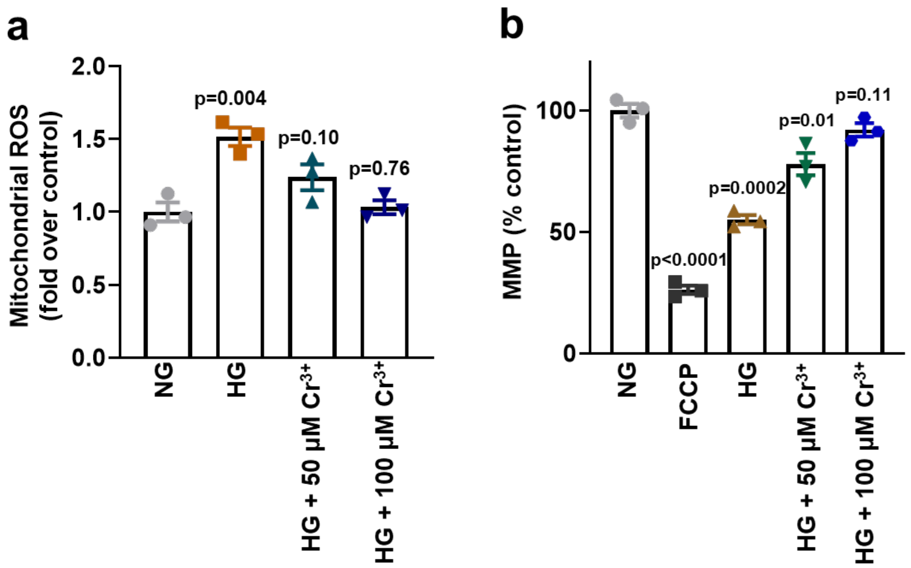

Supplementary Fig. 10 | Effect of $CrCl_3$ treatment on mitochondrial ROS and mitochondrial membrane potential (MMP) in HepG2 cells under high glucose (HG). (**a**) Mitochondrial ROS. n = 3; mean ± SEM. Two-sided Student's *t* test. (**b**) Mitochondrial membrane potential. n = 3; mean ± SEM. Two-sided Student's *t* test. Normal glucose (NG) is used as control. Results are shown as mean ± SEM. Source data are provided as a Source Data file.

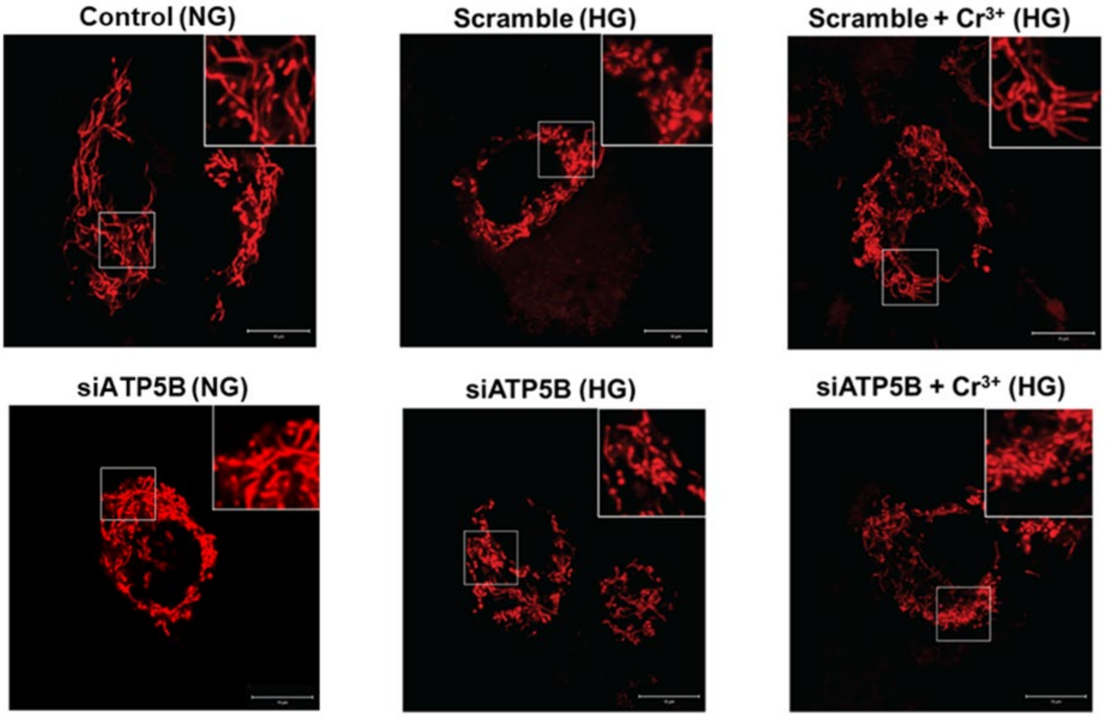

Supplementary Fig. 11 | Cr^{3+} rescues mitochondria from hyperglycaemia-induced fragmentation through targeting ATP5B. Microscopic imaging of wild-type (WT) HepG2 cells, HepG2 cells transfected with negative control siRNA (scramble) or ATP5B siRNA with mitochondria stained by MitoTracker® under normal glucose condition (5.6 mM) (NG), high glucose-induced stress (40 mM, HG) and high glucose-induced stress with Cr^{3+} supplementation (100 µM) (HG + Cr^{3+}) (n = 5). Inset: 2 times enlargement of mitochondria. Scale bars: 5 µm. Source data are presented in the figure.

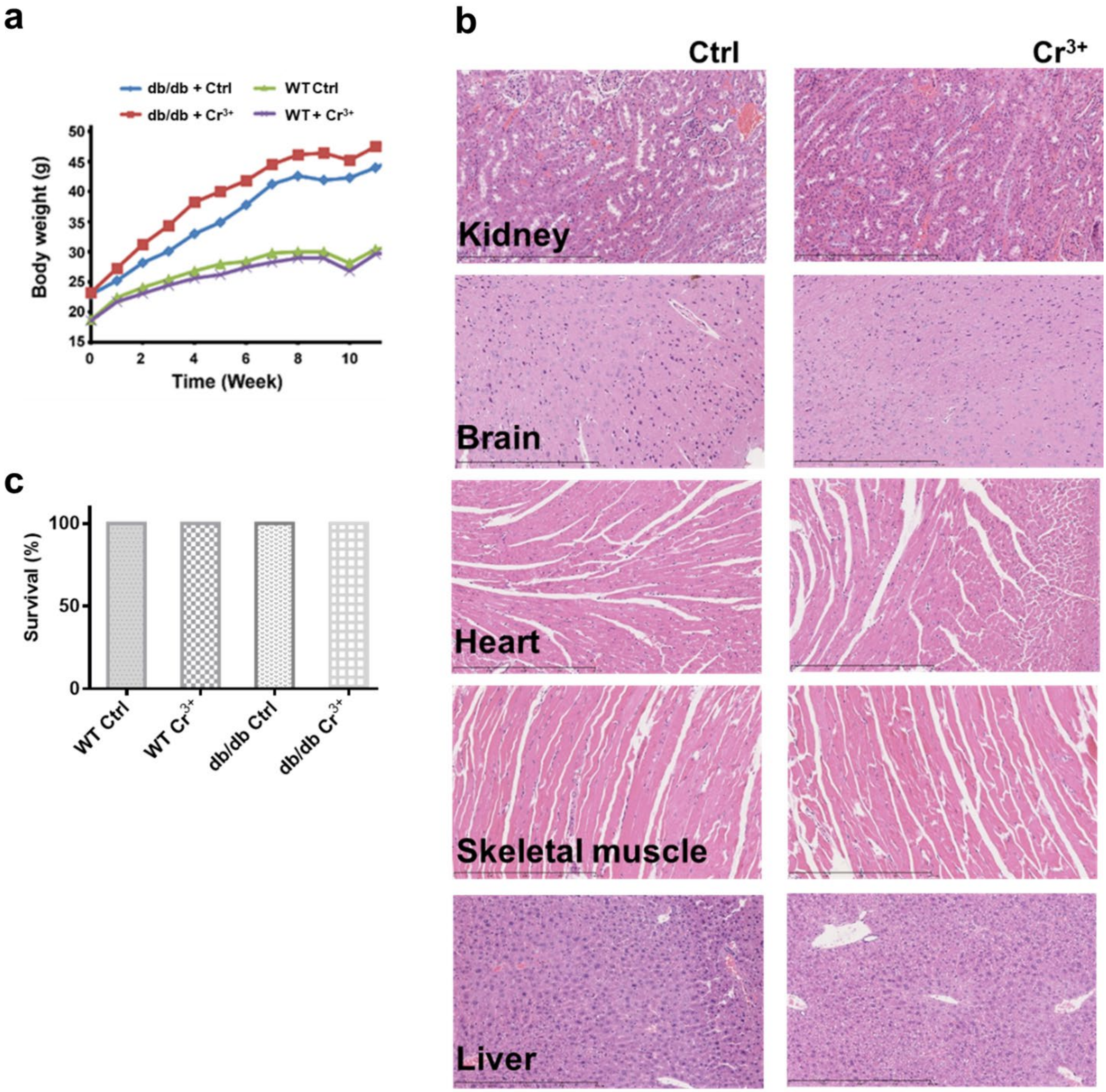

Supplementary Fig. 12 | The body weight/viability of mice with or without treatment of Cr^{3+}. (**a**) Body weight change of db/db and wild-type (WT) mice. Results are shown as mean (n = 6). (**b**) Survival of mice after Cr^{3+} treatment for 10 weeks (n = 6). (**c**) Histologic examination of different organ tissues (n = 6). Hematoxylin-eosin-stained kidney, brain, heart, skeletal muscle, and liver sections from WT mice fed with either a control or Cr^{3+} diet. One representative result of six independent animals is shown. Scale bar: 200 µm. Source data are provided as a Source Data file.

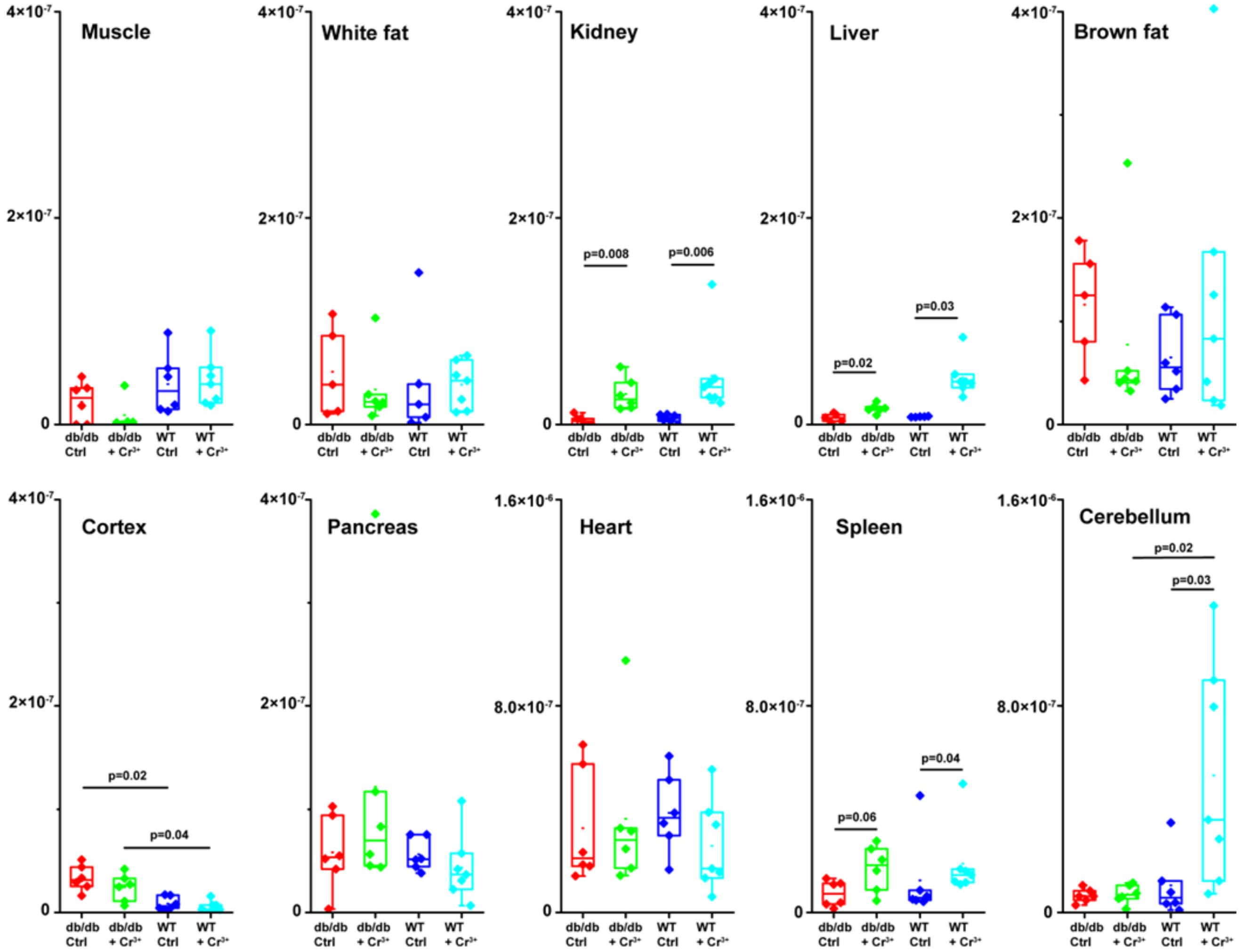

Supplementary Fig. 13 | Cr^{3+} concentration in different organs of mice. Chromium in different organs, including muscle, white fat, kidney, liver, brown fat, cortex, pancreas, heart, spleen, and cerebellum, from mice dosed orally with chromium was quantified by ICP-MS (n = 6). Two-tailed t-test was used for all comparisons between two groups. For box plots, center line, median; box limits, upper and lower boundary, 75% and 25% interquartile ranges respectively; whiskers, maxima and minima; points, all data points. Two-sided Student's *t* test. Source data are provided as a Source Data file.

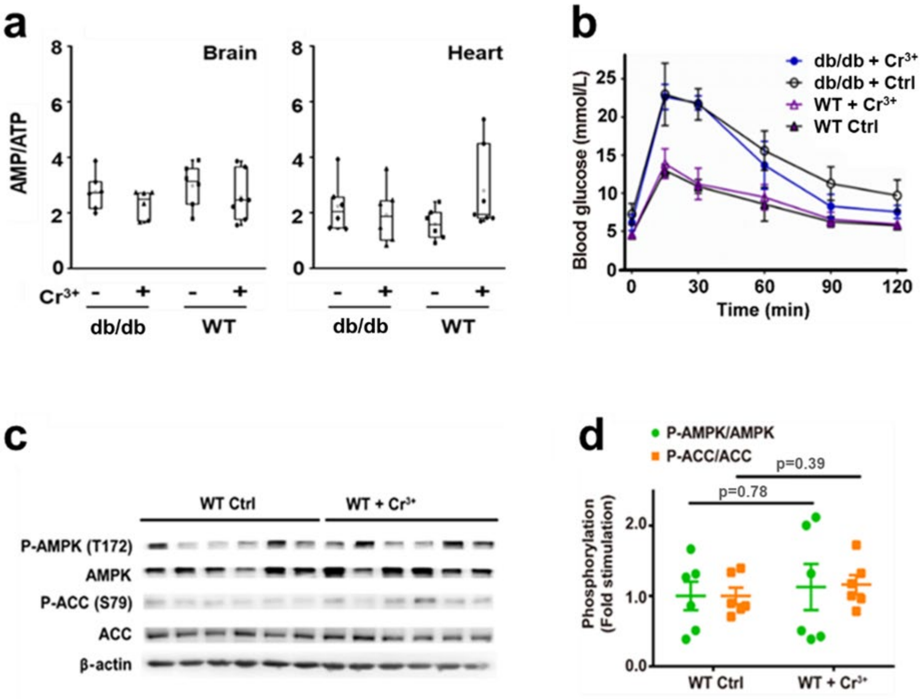

Supplementary Fig. 14 | Cr^{3+} activates AMPK and ACC in db/db mice but not WT mice. (**a**) AMP/ATP ratios in different organs (brain and heart) of mice (n = 6). For box plots, center line, median; box limits, upper and lower boundary, 75% and 25% interquartile ranges respectively; whiskers, max and min; points, all data points. (**b**) Oral Glucose Tolerance Test (OGTT) curve of db/db and WT mice. n = 6; mean ± SEM. (**c**, **d**) Western blot analyses of AMPK, P-AMPK, ACC, and P-ACC in the liver of WT mice with or without treatment of Cr^{3+}. n = 6; mean ± SEM. Two-sided Student's *t* test. Source data are provided as a Source Data file.

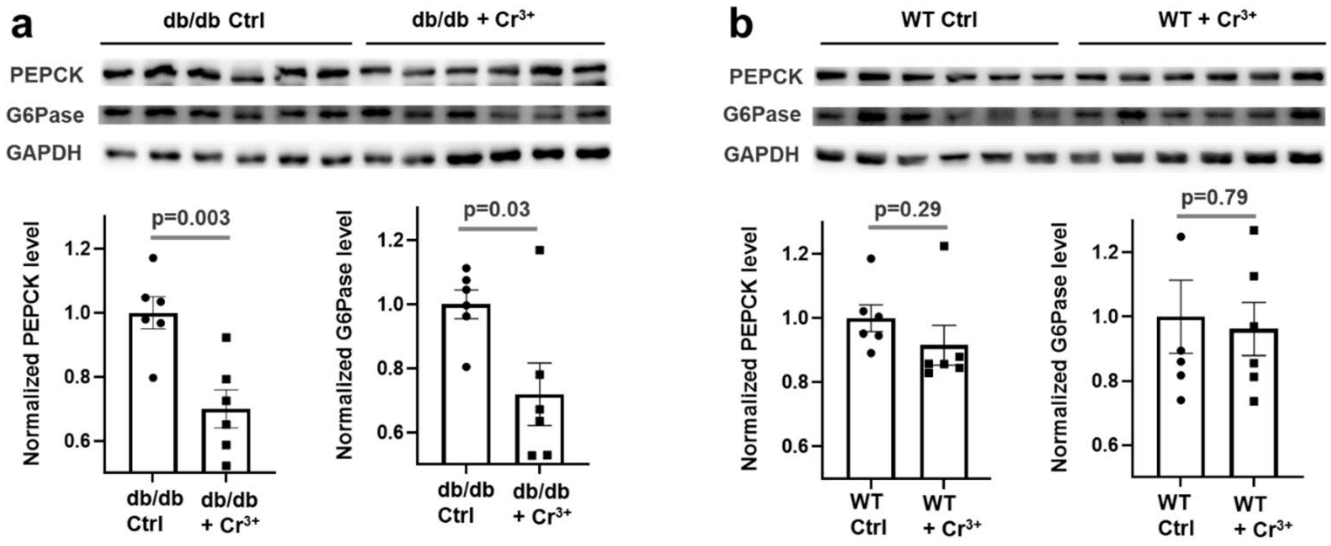

Supplementary Fig. 15 | $CrCl_3$ suppresses hepatic gluconeogenesis in db/db mice but not normal mice. The effect of Cr^{3+} treatment on the expression level of hepatic gluconeogenesis enzymes in db/db (**a**) and (**b**) WT mice. n = 6; mean ± SEM. Two-sided Student's *t* test. Source data are provided as a Source Data file.

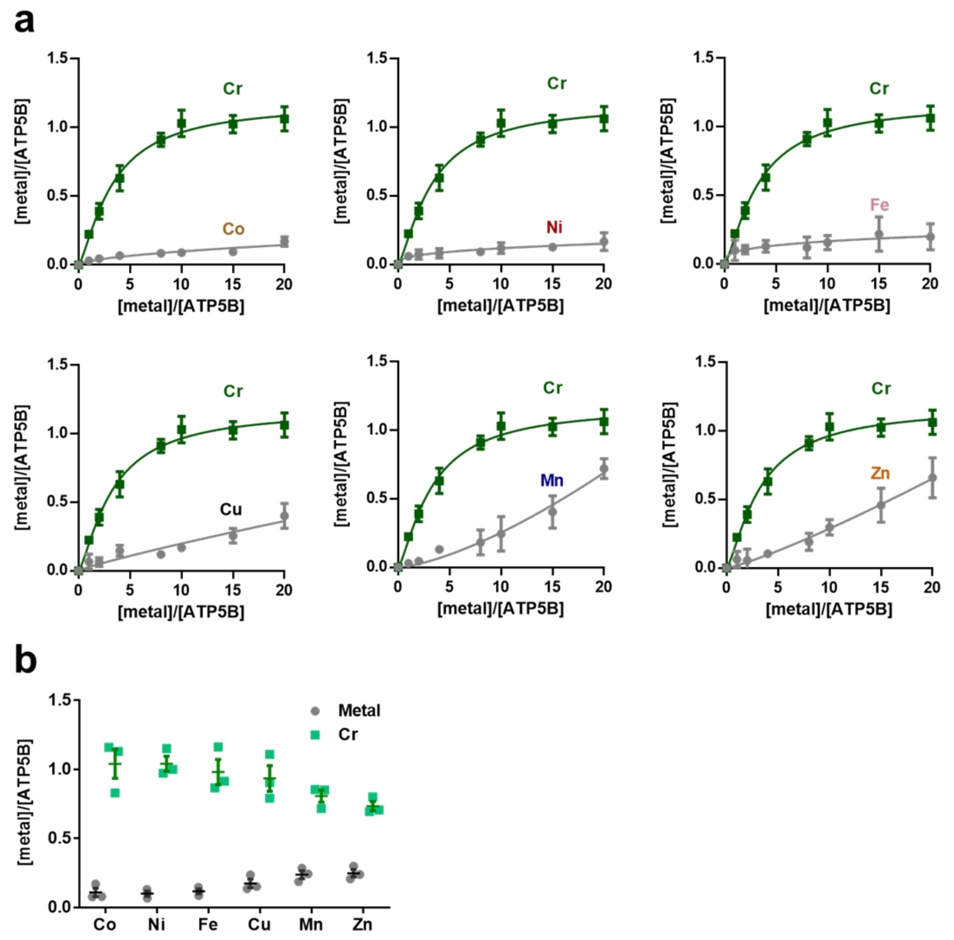

Supplementary Fig. 16 | Binding capability of different transition metal ions to ATP5B. (**a**) Dose-dependent binding of different metal ions to ATP5B. n = 3; mean ± SEM. (**b**) The metal contents in Cr-ATP5B upon the supplementation of different metal ions. n = 3; mean ± SEM. Source data are provided as a Source Data file.

Supplementary Tables

Supplementary Table 1. Summary of Cr^{3+}-associated proteins in HepG2 tracked by Cr^{3+}-NTA-AC in vivo.

**No.**	**Gene**	**Gene product**	**MW/ Da**	**Accession No.**	**Protein Score**	**Total ion score**	**Protein Score C.I.%**	**Peptide count**
1	Hsp60	Mitochondrial heat shock 60 kDa protein	60813.3	gi|189502784	378	270	100	17
2	ATP5B*	ATP synthase subunit beta, mitochondrial precursor	58524.6	gi|32189394	662	476	100	20
3	CLIC1	Chloride intracellular channel protein 1	27247.9	gi|14251209	422	298	100	13
4	PRDX1	Peroxiredoxin 1	10726.5	gi|55959888	64	39	100	3
5	COMT	Catechol-O-methyltransferase	20374.4	gi|33875419	360	278	100	9
6	TXN	Thioredoxin	12014.8	gi|50592994	143	86	100	6
7	H3F3A	H3 histone, family 3A	14157.9	gi|55665435	138	118	100	4
8	ATP5L	ATP synthase subunit g, mitochondrial	11421.2	gi|51479156	60	26	100	4

*ATP synthase is composed of two linked multi-subunit complexes, including the soluble catalytic core F_1 and the membrane-spanning component F_o. ATP5B belongs to the soluble catalytic core F_1 and therefore being observed in the soluble fraction.

Supplementary Table 2. Peptide mass fingerprints of purified Hsp60.

Protein Name		Accession No.	Protein Score	Protein Score C.I. %	Protein MW/Da	Peptide Count
60 kDa heat shock protein, mitochondrial		gi|31542947	276	100	61345.5	15
**Calc. Mass**	**Obs. Mass**	**Peptide Seq.**		**Modification**	**Ion Score**	**C.I. %**
941.6141	941.6689	IGIEIIKR				
960.5109	960.5648	VTDALNATR				
961.4849	961.5546	APGFGDNRK				
1011.5693	1011.6306	GANPVEIRR				
1215.658	1215.7274	NAGVEGSLIVEK				
1344.7158	1344.7906	TVIIEQSWGSPK				
1389.705	1389.7827	GYISPYFINTSK				
1601.7516	1601.8445	CEFQDAYVLLSEK		Carbamidomethyl (C)[1]		
1630.9084	1630.9923	VGEVIVTKDDAMLLK				
1646.9033	1646.9806	VGEVIVTKDDAMLLK		Oxidation (M)[12]		
1684.905	1684.9977	AAVEEGIVLGGGCALLR		Carbamidomethyl (C)[13]	142	100
1684.905	1684.9977	AAVEEGIVLGGGCALLR		Carbamidomethyl (C)[13]		
1729.8466	1729.957	CEFQDAYVLLSEKK		Carbamidomethyl (C)[1]		
1771.8531	1771.9607	CIPALDSLTPANEDQK		Carbamidomethyl (C)[1]		
1914.9265	1915.0348	GQKCEFQDAYVLLSEK		Carbamidomethyl (C)[4]		

Supplementary Table 3. Peptide mass fingerprints of purified ATP5B.

Protein Name		Accession No.	Protein Score	Protein Score C.I. %	Protein MW/Da	Peptide Count
Mitochondrial ATP synthase, H^+ transporting F1, complex beta subunit		gi|89574029	378	100	56524.6	18
**Calc. Mass**	**Obs. Mass**	**Peptide Seq.**		**Modification**	**Ion Score**	**C.I. %**
975.5621	975.6116	IGLFGGAGVGK				
1038.5942	1038.6519	IPVGPETLGR				
1088.635	1088.6943	VVDLLAPYAK				
1191.6732	1191.6135	GVQKILQDYK				
1262.6409	1262.7043	TIAMDGTEGLVR				
1278.6359	1278.6975	TIAMDGTEGLVR		Oxidation (M)[4]		
1385.7094	1385.7805	IMNVIGEPIDER		Oxidation (M)[2]		
1406.681	1406.7484	AHGGYSVFAGVGER			112	100
1435.7539	1435.8184	FTQAGSEVSALLGR				
1439.7893	1439.8538	VALTGLTVAEYFR			66	99.9
1601.8104	1601.8896	VALVYGQMNEPPGAR		Oxidation (M)[8]	15	0
1650.9174	1650.9904	LVLEVAQHLGESTVR			129	100
1780.9625	1781.0431	IMNVIGEPIDERGPIK		Oxidation (M)[2]		
1831.8644	1831.9504	IMDPNIVGSEHYDVAR		Oxidation (M)[2]		
1919.0959	1919.1842	VLDSGAPIKIPVGPETLG			155	100
1988.0334	1988.1268	AIAELGIYPAVDPLDSTSR				
2023.0106	2023.1111	FLSQPFQVAEVFTGHMFG				
2037.1086	2037.2023	ETRLVLEVAQHLGESTVR				
2039.0055	2039.1202	FLSQPFQVAEVFTGHMGK		Oxidation (M)[16]		
2266.0842	2266.1968	IPSAVGYQPTLATDMGT MQER				
2282.0791	2282.1987	IPSAVGYQPTLATDMGT MQER				
2298.074	2298.1912	IPSAVGYQPTLATDMGT MQER		Oxidation (M)[15,18]		

Supplementary Table 4. Peptide mass fingerprints of purified ATP5L.

**Protein Name**		**Accession No.**	**Protein Score**	**Protein Score C.I. %**	**Protein MW/Da**	**Peptide Count**
F1F0-type ATP synthase subunit g		gi|3659901	80	99.98	11379.2	4
**Calc. Mass**	**Obs. Mass**	**Peptide Seq.**		**Modification**	**Ion Score**	**C.I. %**
1151.6055	1151.6708	IVNSAQTGSFK				
1162.5931	1162.6604	LATFWYYAK				
1320.7092	1320.6637	MGQFVRNLVEK				
1587.8854	1587.9659	TPALVNAAVTYSKPR			26	11.078
1587.8854	1587.9659	TPALVNAAVTYSKPR				

Supplementary Table 5. Summary of CETSA results.

**Protein name**	**T_{agg} (ºC)**	**T_{agg} (ºC)**	
	**Ctrl**	**Cr^{3+}**	**Δ T_{agg} (ºC)**
ATP5L	52.9	52.0	0.9
ATP5B	55.5	53.2	2.3
Hsp60	57.2	55.9	1.3
CLIC1	65.3	61.4	3.9
PRDX1	63.2	60.5	2.7
TXN	64.1	60.8	3.3
COMT	56.9	51.7	5.2
H3F3A	59.3	53.8	5.5
