# Supplementary material for: Mitochondrial ATP synthase as a direct molecular target of chromium(III) to ameliorate hyperglycaemia stress
Source: Nat Commun. 2023 Mar 28;14:1738. doi: 10.1038/s41467-023-37351-w (PMC10050403; doi:10.1038/s41467-023-37351-w)
Supplement: Supplementary file 3 — Reporting Summary [file 41467_2023_37351_MOESM3_ESM.pdf]

nature portfolio

Corresponding author(s): Hongzhe Sun

Last updated by author(s): Feb 9, 2023

# Reporting Summary

Nature Portfolio wishes to improve the reproducibility of the work that we publish. This form provides structure for consistency and transparency in reporting. For further information on Nature Portfolio policies, see our Editorial Policies and the Editorial Policy Checklist.

## Statistics

For all statistical analyses, confirm that the following items are present in the figure legend, table legend, main text, or Methods section.

| n/a | Confirmed | |
|---|---|---|
| ☐ | ☒ | The exact sample size (*n*) for each experimental group/condition, given as a discrete number and unit of measurement |
| ☐ | ☒ | A statement on whether measurements were taken from distinct samples or whether the same sample was measured repeatedly |
| ☐ | ☒ | The statistical test(s) used AND whether they are one- or two-sided<br>*Only common tests should be described solely by name; describe more complex techniques in the Methods section.* |
| ☒ | ☐ | A description of all covariates tested |
| ☒ | ☐ | A description of any assumptions or corrections, such as tests of normality and adjustment for multiple comparisons |
| ☐ | ☒ | A full description of the statistical parameters including central tendency (e.g. means) or other basic estimates (e.g. regression coefficient) AND variation (e.g. standard deviation) or associated estimates of uncertainty (e.g. confidence intervals) |
| ☐ | ☒ | For null hypothesis testing, the test statistic (e.g. *F*, *t*, *r*) with confidence intervals, effect sizes, degrees of freedom and *P* value noted<br>*Give P values as exact values whenever suitable.* |
| ☒ | ☐ | For Bayesian analysis, information on the choice of priors and Markov chain Monte Carlo settings |
| ☒ | ☐ | For hierarchical and complex designs, identification of the appropriate level for tests and full reporting of outcomes |
| ☒ | ☐ | Estimates of effect sizes (e.g. Cohen's *d*, Pearson's *r*), indicating how they were calculated |

*Our web collection on statistics for biologists contains articles on many of the points above.*

## Software and code

Policy information about availability of computer code

| | |
|---|---|
| Data collection | Fluorescent and phase contrast images were captured by a Carl Zeiss LSM700 Inverted Confocal Microscope.<br>Immunoreactive bands were visualized by the enhanced chemiluminescence detection system and the intensity of bands was quantified using a model GS-700 Imaging Densitometer (Bio-Rad).<br>The ICP-MS data was collected using ICP-MS (Agilent 7700x). |
| Data analysis | MS data was processed using FlexAnalysis (version 1.2, Bruker Daltonics).<br>Mitochondrial morphology was analyzed using MetaMorph software (version Premier 7.7.0.0 ).<br>The band intensities of western blot were quantified by Image J (V 1.52d).<br>Statistical analysis were performed using Graph Pad Prism software 6 (Graph Pad Software, Inc., La Jolla, CA).<br>ChemBioDraw Ultra (version 14.0.0.117) |

For manuscripts utilizing custom algorithms or software that are central to the research but not yet described in published literature, software must be made available to editors and reviewers. We strongly encourage code deposition in a community repository (e.g. GitHub). See the Nature Portfolio guidelines for submitting code & software for further information.

## Data

Policy information about availability of data

All manuscripts must include a data availability statement. This statement should provide the following information, where applicable:

- Accession codes, unique identifiers, or web links for publicly available datasets
- A description of any restrictions on data availability
- For clinical datasets or third party data, please ensure that the statement adheres to our policy

The mass spectrometry proteomics data have been deposited to the ProteomeXchange Consortium via the PRIDE partner repository with the dataset identifier PXD027305 (https://www.ebi.ac.uk/pride/archive/projects/PXD027305). The protein-protein interaction network of Cr(III)-associated proteins was obtained and visualized as confidence view via STRING 9.1 (www. string-db.org). All data supporting the findings of this study are available in a publicly accessible repository or in the Source Data file. The source data are provided with this paper. A reporting summary for this article is available as a Supplementary Information file.

## Human research participants

Policy information about studies involving human research participants and Sex and Gender in Research.

| | |
|---|---|
| Reporting on sex and gender | Not applicable |
| Population characteristics | Not applicable |
| Recruitment | Not applicable |
| Ethics oversight | Not applicable |

Note that full information on the approval of the study protocol must also be provided in the manuscript.

# Field-specific reporting

Please select the one below that is the best fit for your research. If you are not sure, read the appropriate sections before making your selection.

☒ Life sciences ☐ Behavioural & social sciences ☐ Ecological, evolutionary & environmental sciences

For a reference copy of the document with all sections, see nature.com/documents/nr-reporting-summary-flat.pdf

# Life sciences study design

All studies must disclose on these points even when the disclosure is negative.

| | |
|---|---|
| Sample size | Preliminary experiments were performed when possible to determine requirements for sample size. Sample size sufficiency was determined by preliminary data or discussion. For statistical significance, the sample size was always independently performed three or more times. |
| Data exclusions | No data were excluded from the analysis. |
| Replication | Unless specified, all experiments were subjected to three biological replicates and two technique replicates, and all attempts at replication were successful. |
| Randomization | Samples were allocated randomly into experimental groups. |
| Blinding | We were blinded to group allocation. |

# Reporting for specific materials, systems and methods

We require information from authors about some types of materials, experimental systems and methods used in many studies. Here, indicate whether each material, system or method listed is relevant to your study. If you are not sure if a list item applies to your research, read the appropriate section before selecting a response.

## Materials & experimental systems

| n/a | Involved in the study |
|---|---|
| ☐ | ☒ Antibodies |
| ☐ | ☒ Eukaryotic cell lines |
| ☒ | ☐ Palaeontology and archaeology |
| ☐ | ☒ Animals and other organisms |
| ☒ | ☐ Clinical data |
| ☒ | ☐ Dual use research of concern |

## Methods

| n/a | Involved in the study |
|---|---|
| ☒ | ☐ ChIP-seq |
| ☒ | ☐ Flow cytometry |
| ☒ | ☐ MRI-based neuroimaging |

# Antibodies

Antibodies used

Anti-AMPK, Abcam, ab32047 (Y365, monoclonal)
Anti-phospho-AMPK (Thr172), Abcam, ab133448 (EPR5683, monoclonal)
Anti-ACC, Abcam, ab45174 (EP687Y, monoclonal)
Anti-phosphoacetyl CoA carboxy lase (ACC, Ser 79), Abcam, ab31931 (Polyclonal)
Anti-GAPDH, Abcam, ab181602, (EPR16891, monoclonal)
Anti-ATPB, Abcam, ab14730, (3D5, monoclonal)
Anti-ATP5C1, Abcam, ab119686, (2A1AA11, monoclonal)
Anti-Hsp60, Abcam, ab46798, (Polyclonal)
Anti-β-actin, Abcam, ab1801, (Polyclonal)
Anti-PEPCK, Abclonal, A4466, (ARC1017, monoclonal)
Anti-G6Pase, Abclonal, A20193, (Polyclonal)
Anti-GCK, Abclonal, A5170, (ARC1226, monoclonal)
Anti-PFKL, Abclonal, A7708, (Polyclonal)
Anti-CLIC1, abclonal, A6363, (Polyclonal)
Anti-PRDX1, abclonal, A1842, (Polyclonal)
Anti-TXN, abclonal, A0537, (Polyclonal)
Anti-COMT, abclonal, A1294, (Polyclonal)
Anti-H3F3A, abclonal, A10220, (Polyclonal)
Anti-rabbit IgG, HRP-linked antibody, Cell Signaling Technology, 7074s
Anti-mouse IgG, HRP-linked antibody, Cell Signaling Technology, 7076s

Validation

Anti-AMPK, Abcam, ab32047: A Cr3+-mediated time- and dose-dependent increases in AMPK phosphorylation were observed compared to the control group (Fig. 2d and 2e). The antibody guarantee covers the use of the antibody for WB. Species reactivity: Mouse, Rat and Human. Clonality: monoclonal. Host: Rabbit. Conjugate: Unconjugated. https://www.abcam.com/ampk-alpha-1-antibody-y365-ab32047.html

Anti-phospho-AMPK (Thr172), Abcam, ab133448: A Cr3+-mediated time- and dose-dependent increases in AMPK phosphorylation were observed compared to the control group (Fig. 2d and 2e). The antibody guarantee covers the use of the antibody for WB. Species reactivity: Mouse, Rat, Human and Drosophila melanogaster. Clonality: monoclonal. Host: Rabbit. Conjugate: Unconjugated. https://www.abcam.cn/ampk-alpha-1-phospho-t183--ampk-alpha-2-phospho-t172-antibody-epr5683-ab133448.html

Anti-ACC, Abcam, ab45174: A Cr3+-mediated time- and dose-dependent increases in the phosphorylation of ACC were observed compared to the control group (Fig. 2d and 2e). The antibody guarantee covers the use of the antibody for WB. Species reactivity: Mouse, Rat and Human. Clonality: monoclonal. Host: Rabbit. Conjugate: Unconjugated. https://www.abcam.cn/acetyl-coenzyme-a-carboxylase-antibody-ep687y-ab45174.html

Anti-phosphoacetyl CoA carboxy lase (ACC, Ser 79), Abcam, ab31931: A Cr3+-mediated time- and dose-dependent increases in the phosphorylation of ACC were observed compared to the control group (Fig. 2d and 2e). The antibody guarantee covers the use of the antibody for WB. Species reactivity: Human. Clonality: Polyclonal. Host: Rabbit. Conjugate: Unconjugated. https://www.abcam.cn/acetyl-coenzyme-a-carboxylase-phospho-s79-antibody-ab31931.html

Anti-GAPDH, Abcam, ab181602: GAPDH served as the internal standard (Fig. 2d, 2e, 2f and 4b). The antibody guarantee covers the use of the antibody for WB. Species reactivity: Mouse, Rat, Chicken, Human, Zebrafish, African green monkey, Xenopus tropicalis. Clonality: monoclonal. Host: Rabbit. Conjugate: Unconjugated. https://www.abcam.cn/gapdh-antibody-epr16891-loading-control-ab181602.html

Anti-ATPB, Abcam, ab14730: CrCl3 treatment resulted in apparent aggregation temperature shift for ATPB (Fig. 1g). The antibody guarantee covers the use of the antibody for WB. Species reactivity: Mouse, Rat, Cow, Human, Caenorhabditis elegans. Clonality: monoclonal. Host: Mouse. Conjugate: Unconjugated. https://www.abcam.cn/atpb-antibody-3d5-mitochondrial-marker-ab14730.html

Anti-ATP5C1, Abcam, ab119686: CrCl3 treatment resulted in apparent aggregation temperature shift for ATP5C1 (Fig. 1g). The antibody guarantee covers the use of the antibody for WB. Species reactivity: Mouse, Rat, Cow, Human. Clonality: monoclonal. Host: Mouse. Conjugate: Unconjugated. https://www.abcam.cn/atp5c1-antibody-2a1aa11-ab119686.html

Anti-Hsp60, Abcam, ab46798: CrCl3 treatment resulted in apparent aggregation temperature shift for Hsp60 (Fig. 1g). The antibody guarantee covers the use of the antibody for WB. Species reactivity: Mouse, Rat, Human, Pig. Clonality: Polyclonal. Host: Mouse. Conjugate: Unconjugated. https://www.abcam.cn/hsp60-antibody-ab46798.html

Anti-β-actin, Abcam, ab1801: β-actin served as the internal standard (Fig. 4i). The antibody guarantee covers the use of the antibody for WB. Species reactivity: Mouse, Rat, Human. Clonality: Polyclonal. Host: Rabbit. Conjugate: Unconjugated. https://www.abcam.cn/actin-antibody-loading-control-ab1801.html

Anti-PEPCK, Abclonal, A4466: CrCl3 treatment resulted in significant suppression of gluconeogenesis enzyme PEPCK (Fig. 4b and 4c). The antibody guarantee covers the use of the antibody for WB. Species reactivity: Mouse, Rat, Human. Clonality: Monoclonal. Host: Rabbit. Conjugate: Unconjugated. https://abclonal.com.cn/catalog/A4466

Anti-G6Pase, Abclonal, A20193: CrCl3 treatment resulted in significant suppression of gluconeogenesis enzyme G6Pase (Fig. 4b and 4c). The antibody guarantee covers the use of the antibody for WB. Species reactivity: Mouse. Clonality: Polyclonal. Host: Mouse. Conjugate: Unconjugated. https://abclonal.com.cn/catalog/A20193

Anti-GCK, Abclonal, A5170: CrCl3 treatment resulted in significant upregulation of glycolysis enzyme GCK (Fig. 4b and 4c). The antibody guarantee covers the use of the antibody for WB. Species reactivity: Mouse, Rat, Human. Clonality: Monoclonal. Host: Rabbit. Conjugate: Unconjugated. https://abclonal.com.cn/catalog/A5170

Anti-PFKL, Abclonal, A7708: CrCl3 treatment resulted in significant upregulation of glycolysis enzyme PFKL (Fig. 4b and 4c). The antibody guarantee covers the use of the antibody for WB. Species reactivity: Mouse, Rat, Human. Clonality: Polyclonal. Host: Rabbit. Conjugate: Unconjugated. https://abclonal.com.cn/catalog/A7708

Anti-CLIC1, abclonal, A6363: CrCl3 treatment resulted in apparent aggregation temperature shift for CLIC1 (Supplementary Fig. 4b). The antibody guarantee covers the use of the antibody for WB. Species reactivity: Mouse, Rat, Human. Clonality: Polyclonal. Host: Rabbit. Conjugate: Unconjugated. https://abclonal.com.cn/catalog/A6363

Anti-PRDX1, abclonal, A1842: CrCl3 treatment resulted in apparent aggregation temperature shift for PRDX1 (Supplementary Fig. 4b). The antibody guarantee covers the use of the antibody for WB. Species reactivity: Mouse, Human. Clonality: Polyclonal. Host: Rabbit. Conjugate: Unconjugated. https://abclonal.com.cn/Datasheet/Antibodies/A1842.pdf?v=1647408466

Anti-TXN, abclonal, A0537: CrCl3 treatment resulted in apparent aggregation temperature shift for TXN (Supplementary Fig. 4b). The antibody guarantee covers the use of the antibody for WB. Species reactivity: Human. Clonality: Polyclonal. Host: Rabbit. Conjugate: Unconjugated. https://abclonal.com.cn/Datasheet/Antibodies/A0537.pdf?v=1621839583

Anti-COMT, abclonal, A1294: CrCl3 treatment resulted in apparent aggregation temperature shift for COMT (Supplementary Fig. 4b). The antibody guarantee covers the use of the antibody for WB. Species reactivity: Human, Mouse. Clonality: Polyclonal. Host: Rabbit. Conjugate: Unconjugated. https://abclonal.com.cn/catalog/A1294

Anti-H3F3A, abclonal, A10220: CrCl3 treatment resulted in apparent aggregation temperature shift for H3F3A (Supplementary Fig. 4b). The antibody guarantee covers the use of the antibody for WB. Species reactivity: Human. Clonality: Polyclonal. Host: Rabbit. Conjugate: Unconjugated. https://abclonal.com.cn/catalog/A10220

## Eukaryotic cell lines

Policy information about cell lines and Sex and Gender in Research

| | |
|---|---|
| Cell line source(s) | HepG2 (Catalog No. HB-8065), Hela (Catalog No. CRM-CCL-2) and C2C12 myoblast cell line (Catalog No. CRL-1772) were purchased from American Type Culture Collection (ATCC). |
| Authentication | Cell lines were authenticated by short tandem repeat analysis and/or in vitro differentiation. |
| Mycoplasma contamination | Mycoplasma contamination was routinely checked and negative results were obtained. |
| Commonly misidentified lines (See ICLAC register) | No commonly misidentified cell lines were used in the study. |

## Animals and other research organisms

Policy information about studies involving animals; ARRIVE guidelines recommended for reporting animal research, and Sex and Gender in Research

| | |
|---|---|
| Laboratory animals | C57BL/Ks (BKS) wild-type and type II diabetes model mice db/db were purchased from Cavens Lab Animal, Inc. of age 5 weeks old at the beginning of experiment. The inbred mouse strain BKS carrying a mutation of the leptin receptor lepr. (BKS-db) is a classic mouse model of type 2 diabetes. All animals are male mice.<br>The mice were housed in microisolator cages under temperature of 22–24 °C, relative humidity of 40%-70% and light control conditions of 12-h/12-h light/dark cycle at the Department of Laboratory Animal Science, Research Center for Eco-Environmental Sciences, Chinese Academy of Sciences, Beijing, China. The experimental procedures were approved by Committee of Scientific Research in Research Center for Eco-Environmental Sciences, Chinese Academy of Sciences. All animals were treated humanely to minimize their sufferings during the experiments. |
| Wild animals | The study did not involve wild animals. |

| | |
|---|---|
| Reporting on sex | All animals used in our study were males. |
| Field-collected samples | The study did not involve samples collected from the field. |
| Ethics oversight | All experiments were performed in accordance with the Animal Use Protocol approved by the Animal Care and Use Committee of Peking University Health Science Center. |

Note that full information on the approval of the study protocol must also be provided in the manuscript.
